# Impact of Goji Berries (*Lycium barbarum*) Supplementation on the Energy Homeostasis of Rabbit Does: Uni- and Multivariate Approach

**DOI:** 10.3390/ani10112000

**Published:** 2020-10-30

**Authors:** Laura Menchetti, Giulio Curone, Egon Andoni, Olimpia Barbato, Alessandro Troisi, Bernard Fioretti, Angela Polisca, Michela Codini, Claudio Canali, Daniele Vigo, Gabriele Brecchia

**Affiliations:** 1 Department of Agricultural and Agri-food Sciences and Technologies, University of Bologna, Viale Fanin 46, 40138 Bologna, Italy; laura.menchetti7@gmail.com; 2 Department of Veterinary Medicine, University of Perugia, Via San Costanzo 4, 06126 Perugia, Italy; angela.polisca@unipg.it (A.P.); claudio.canali@unipg.it (C.C.); 3 Department of Veterinary Medicine, University of Milano, Via dell’Università 6, 26900 Lodi, Italy; giulio.curone@unimi.it (G.C.); daniele.vigo@unimi.it (D.V.); 4 Faculty of Veterinary Medicine, Agricultural University of Albania, Rr Paisi Vodica, Koder, 1029 Kamez, Albania; eandoni@ubt.edu.al; 5 School of Biosciences and Veterinary Medicine, University of Camerino, Via 9 Circonvallazione 93/95-62024 Matelica, Italy; alessandro.troisi75@gmail.com; 6 Department of Chemistry, Biology and Biotechnologies, University of Perugia, Via Elce di Sotto 8, 06123 Perugia, Italy; bernard.fioretti@unipg.it; 7 Department of Pharmaceutical Sciences, University of Perugia, Via A. Fabretti 48, 06123 Perugia, Italy; michela.codini@unipg.it

**Keywords:** goji berries, rabbit, insulin resistance, leptin, non-esterified fatty acids, pregnancy, lactation, body condition score, principal component analysis

## Abstract

**Simple Summary:**

The energy balance during the reproduction cycle is a problematic issue for livestock species because it has consequences not only on animal welfare but also on the profitability of the farm. The adoption of new nutritional strategies could improve both of these aspects. In the present study, the supplementation with goji berries was proposed and evaluated on the rabbit, which is both a livestock animal and a useful animal model. Goji berry is the fruit of *Lycium barbarum* that is a natural resource made up of several compounds with biological activities and their consumption could be beneficial for the health and the general well-being of humans and animals. Its effect on several hormones and metabolites involved on energy balance of rabbit doe were evaluated by using both uni- and multivariate approach. Our finding, in addition to describing the intricate relationships between body conditions, hormones and metabolites during pregnancy and lactation, suggested that the supplementation with goji berry in the rabbit diet at low percentage could improve some aspects of energy metabolism and, in particular, doe’s insulin sensitivity. Conversely, the intake of high doses of goji raises concerns due to the risk of excessive fattening and worsening of insulin resistance.

**Abstract:**

This study examined the effects of goji berries dietary supplementation on the energetic metabolism of doe. Thirty days before artificial insemination, 75 New Zealand White does were assigned to three different diets: commercial standard diet (C) and supplemented with 1% (LG) and 3% (HG) of goji berries, respectively. Body conditions, hormones and metabolites were monitored until weaning. Body weight and BCS were higher in HG than C (*p* < 0.05). LG showed lower T3/T4 ratio and cortisol concentrations (*p* < 0.05) and tended to have lower indices of insulin resistances (*p* < 0.1) than HG. Compared to control, leptin was higher in HG at AI (*p* < 0.01) and in LG during lactation (*p* < 0.05). Two principal components were extracted by multivariate analysis describing the relationships between (1) non-esterified fatty acids, insulin and glucose levels, and (2) body conditions and leptin metabolism. The first component highlighted the energy deficit and the insulin resistance of the does during pregnancy and lactation. The second one showed that leptin, body weight and Body Condition Score (BCS) enhance as levels of goji berries in the diet increase. Thus, the effects of goji supplementation are dose-dependent: an improvement on energy metabolism was achieved with a low-dose while the highest dose could determine excessive fattening and insulin resistance in does.

## 1. Introduction

Recently, research has turned towards natural products, rich in compounds with high biological activity that have favourable effects on health with low production expenses and reduced side effects for the prevention and treatment of various pathologies. In this contest, goji berry, the fruit of *Lycium barbarum* L., consumed in several Asian countries for a long time as a traditional tonic food and natural herbal remedy, has attracted a lot of attention also in Western countries in the last decades [1,2]. In fact, there are a lot of evidences showing that the berry has numerous potential beneficial effects for the general well-being of the individual and for the prevention and treatment of numerous pathologies [1,2,3,4]. Beside several biological active compounds such as carotenoids, vitamins (riboflavin, thiamin and ascorbic acid) and flavonoids, goji berry is primarily rich in polysaccharides [1] which are responsible for the main beneficial pharmacological effects of the fruit both in vitro [5,6,7,8] and in vivo in various laboratory animal species [9,10] and in clinical trials in humans [11,12]. The effects of the goji berries are mainly studied in laboratory animals such as mice and rats [9,10,13] and only a few trials were conducted using the rabbit [14,15,16] although it is considered a useful experimental animal model [17,18,19,20,21,22]. Moreover, only a limited number of researches evaluated the effects of goji berry on the reproductive and productive performance, other than on the quality of meat, in livestock animals, rabbits included [15,16,23,24,25].

During pregnancy, the energy demands increase to favor the growth of fetuses and prepare the mother to the subsequent lactation. For these reasons, some changes in the energy homeostasis are necessary. In fact, during this physiological status, the pregnant animal enhances the feed intake and the mobilization of the body reserves as an adaption mechanisms to the modification in energetic request [26,27,28]. Different hormones (insulin, leptin, T3, T4 and cortisol) and metabolites such as glucose and non-esterified fatty acids (NEFA) are implicated in maintaining energy homeostasis during pregnancy in different species [29,30,31,32]. To our knowledge, no studies have evaluated the impact of the goji berries enriched diet on the hormonal control of the energetic homeostasis during pregnancy of the rabbit does.

Therefore, the aims of this research were to evaluate the effects of the integration of the rabbit diet with goji berries at two different concentrations, 1% and 3%, on the body conditions as well as on the levels of several metabolic hormones and metabolites involved in energy homeostasis in pregnant and lactating rabbits does. First, the patterns of these parameters in the three groups was assessed individually using a univariate approach. Subsequently, a multivariate approach was used to identify the main hormonal and metabolic profiles and the effects of goji berries inclusion in the feed on these frameworks.

## 2. Materials and Methods

The study was performed at the facilities of the Faculty of Veterinary Medicine, of the Agricultural University of Tirana, Albania. The experimental protocol was in agreement with the national rules on the use of livestock animal for experimental and other scientific purposes, their handling, protection, and welfare. Every effort has been made to reduce animal discomfit and to use only the number of animals sufficient to produce valid results.

### 2.1. Animals and Experimental Design

Seventy-five New Zealand White nulliparous does were maintained in single cage in controlled environmental conditions: temperature ranged from +18 to +23 °C, relative humidity being from 60% to 75%, and the lighting schedule of 16 L:8 D. Rabbit does, on the base of a random chose, were assigned to three groups depending by the diet administered (*n* = 25/group): Control (C), 1% goji (LG) and 3% goji (HG). Does were fed with commercial feed (control) or the same feed integrated with 1% and 3% of goji berries in LG and HG, respectively (Table 1 [16]). The animals were undergoing artificial insemination (AI) after 30 days of nutritional adaptation to the experimental dietary regime. During this period, they received 150 g/d of feed, while after AI, the rabbits had ad libitum access to feed. Water was always administered ad libitum. An injection of 0.8 μg of synthetic GnRH (Receptal, Hoechst-Roussel Vet, Milan, Italy) just before AI was used to induce the ovulation [33]. AI was executed with 0.5 mL of diluted fresh semen. Pregnancy was determined by abdominal palpation 10 days after AI. Eleven does for each group were monitored during pregnancy and lactation until weaning. Lactation was controlled, the does had access to the nest once a day for ten minutes until day 18 post-partum. The nest was open on the 19th day of lactation. Weaning occurred at 35 days of age separating the little rabbits from their mothers.

### 2.2. Body Conditions

During the experimental period, the feed intake was registered daily while body weight (BW) and body condition score (BCS) were recorded weekly, between 7:30 and 9:00 AM, from time 0 (before the administration of the three experimental diets) until day 35 post-partum (weaning). Weights and BCS were also measured on the day of the IA. The aggregated BCS (from 0 to 4) was obtained by summing the score (0–2) estimated for the rump and the loin [28].

### 2.3. Hormone and Metabolite Assays

Blood samples were collected to evaluate the metabolic hormones (insulin, leptin, T3, T4 and cortisol) and metabolites (glucose and NEFA) concentrations. These were withdrawn at the time 0 (basal), at AI, post-partum, 20th day of lactation (top lactation), and at the post-weaning. The samples were extracted from the central ear vein into tubes containing EDTA, and instantly centrifuged at 3000× *g* for 20 min; subsequently, plasma was frozen and stored until evaluated for hormones and metabolites. Radioimmunological procedures (RIA) were used to evaluate plasma cortisol, insulin, leptin, triiodothyronine (T3) and thyroxine (T4) concentrations as previously reported [26,27,33]. Leptin concentrations were determined by using the multispecies leptin kit based on a double antibody RIA method (Linco Research Inc., St. Charles, MO, USA). The intra- and inter-assay coefficients of variations were 3.4% and 8.7%, respectively while the limit of sensitivity was 1.0 ng/mL.

A porcine insulin RIA kit was used to quantify the plasma levels of insulin making use of double antibody/PEG technique (Linco Research Inc., Saint Charles, MO, USA). A purified recombinant human insulin was used to produce the standard curve as well as labelled antigen while the antibody against the porcine insulin (antiserum) was produced in the guinea pig. The minimum level of insulin detected by the kit was 2 μU/mL. The coefficients of variations were 6.8% for the intra-assay and 9.2% for the interassay.

Thyroid hormones (T3 and T4) were assayed using the procedure reported by the manufacturer on the brochure of the kit (Immunotech, Prague, Czech Republic). The sensitiveness of the analysis was 0.26 nmol/L for T3 and 10.63 nmol/L for T4. The coefficients of variations of T3 were 6.3% for the intraassay and 7.7%, inter-assay; whereas, for T4 were 3.29% for the intraassay and 7.53% for the inter-assay.

CORT kit was used to determine the cortisol plasma concentrations (Immunotech, Prague, Czech Republic). The lower level of cortisol detected by the kit was 2.5 nM and intra-assay and inter-assay coefficients of variations were 5.8% and 9.2%, respectively.

The metabolites glucose and NEFA were assayed by colorimetric methods according to Menchetti et al. [31], and García-García et al. [35] respectively. The NEFA plasma concentrations were evaluated using a colorimetric assay from Wako that use two enzymatic reactions (NEFA-C, Wako Chemicals GmbH, Neuss, Germany), and based on the ability of NEFA to acylate coenzyme A in the presence of CoA synthetase.

A glucose Infinity kit from Sigma (Sigma Diagnostic Inc., St. Louis, MO, USA), that take advantage of the glucose oxidase method, was used to assess the glucose plasma levels.

Finally, the homeostasis model assessment for insulin resistance (HOMA) was used as index of insulin-resistance and it was calculated using a formula previously reported [27,31]: [insulin concentration × (glucose concentration/18)]/22.5. Insulin resistance (low insulin sensibility) is evident when the HOMA-IR is high while low HOMA-IR values are expression of high insulin sensitivity.

### 2.4. Statistical Considerations and Data Analysis

Statistical analyses were performed with SPSS Statistics version 25 (IBM, SPSS Inc., Chicago, IL, USA). We defined *p* ≤ 0.05 as significant and a *p* value between 0.1 and 0.05 as a trend.

First, data were analysed by a univariate approach using the Linear Mixed Model. The models evaluated the effects of the Group (three levels: C, LG or HG), Time (as days, depending on the dependent variable) and Group × Time interaction. The Time was included as a repeated factor. The Sidak method was used for multiple comparisons. We verified assumptions and outliers by using diagnostic graphics. Results were expressed as estimated marginal means ± standard error (SE) but raw data were presented in figures.

Then, the principal component analysis (PCA) was used to describe relationships between body condition, hormones, and metabolites and convert the numerous variables into a few profiles describing the hormonal and metabolic framework. The very low or very high correlations between variables were checked using correlation matrices in order to identify suitable variables for the PCA [36,37,38]. Moreover, the Determinant of the correlation matrix and the Bartlett’s test of Sphericity were also used. The sampling adequacy was tested using a Kaiser–Meyer–Olkin (KMO) value >0.6 as index of adequate factorability. The number of components (PCs) to retain was chosen according to the Kaiser’s criterion (eigenvalues > 1) and Varimax rotation was applied. The interpretation of PCs was mainly based on items having loadings greater than |0.4|. Then, the scores were calculated for each PC using regression techniques to create two new variables (PC1 and PC2) as linear combinations of the indices of body conditions, hormones, and metabolites.

Finally, the effect of percentage of goji berries inclusion on these PCs was evaluated using Generalized Linear Models (GLMs). Normal and Identity were set as the probability distribution and the link function, respectively, while the Time was included as Within-Subject Effects. In the GLMs, the two PCs were evaluated separately as dependent variables and the percentage of goji berries inclusion was included as predictor [16]. In addition, the number of days of supplementation was included as covariate. Results were expressed as b coefficient with SE and *p* value from Wald Chi-square statistics [16,39].

The G*Power^®^ software was used to calculate the achieved power [40]. An F test was chosen for this power analysis by setting α = 0.05 and the effect size = 0.25 (medium effect size) [41]. For the 3 experimental groups (C, LG or HG) and the five repeated measures (basal, AI, post-partum, 20th day of lactation, and post-weaning), a power of 90.5% was reached using a total sample size of 33 animals.

## 3. Results

### 3.1. Univariable Approach

#### 3.1.1. Body Conditions

**Figure 1:**
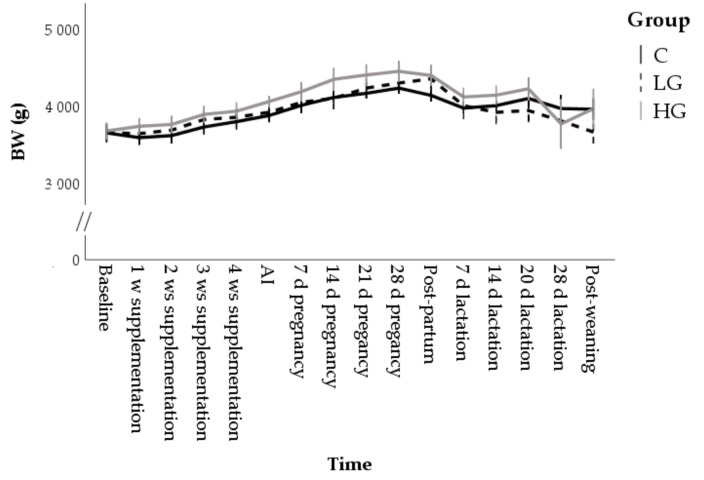
Changes in the body weight (BW) during nutrition adaptation and productive cycle of rabbit does. C = control diet; LG = diet supplemented with 1% of goji berries; HG = diet supplemented with 3% of goji berries. AI = artificial insemination.

BW was influenced by time (F = 9.59; *p* < 0.001) and group (F = 5.43; *p* = 0.005). In supplemented rabbits, the BW increase was early, starting from the first week of pregnancy in LG (4044 ± 128 g; *p* < 0.05) and from AI in HG (4054 ± 88 g; *p* < 0.01) groups. After pregnancy, it returned at baseline values after one week of lactation in C (3969 ± 151 g) and LG (4000 ± 128 g) while, in the HG group, it remained elevated up to 20 d of lactation (4220 ± 139 g) (Figure 1).

**Figure 2:**
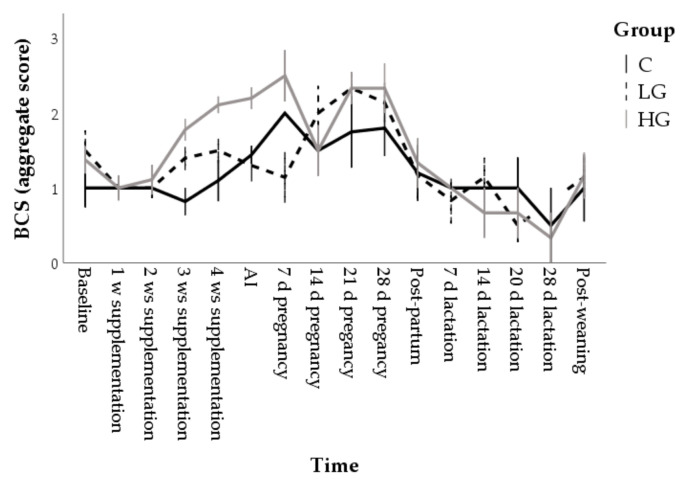
Changes in the body condition score (BCS) during nutrition adaptation and productive cycle of rabbit does. C = control diet; LG = diet supplemented with 1% of goji berries; HG = diet supplemented with 3% of goji berries. AI = artificial insemination.

The aggregate BCS showed significant effects for time (F = 9.51; *p* < 0.001), group (F = 4.19; *p* = 0.016) and their interaction (F = 1.82; *p* = 0.007). Marginal means were 1.1 ± 0.1, 1.3 ± 0.1, and 1.5 ± 0.1 in C, LG, and HG, respectively. HG group showed higher BCS values than C starting from week 3 of supplementation, (*p* < 0.001; Figure 2). From mid-pregnancy onwards, the differences were no longer significant, perhaps also due to the high variability of the data.

Food intake did not differ between groups during pregnancy (144 ± 2 g/d, 146 ± 2 g/d, and 148 ± 3 g/d for C, LG, and HG, respectively; F = 0.78; *p* = 0.459) while it was greater in the control group than supplemented rabbits during lactation (320 ± 2 g/d, 301 ± 2 g/d, and 287 ± 3 g/d for C, LG, and HG, respectively; F = 42.00; *p* < 0.001).

#### 3.1.2. Hormone and Metabolite

**Figure 3:**
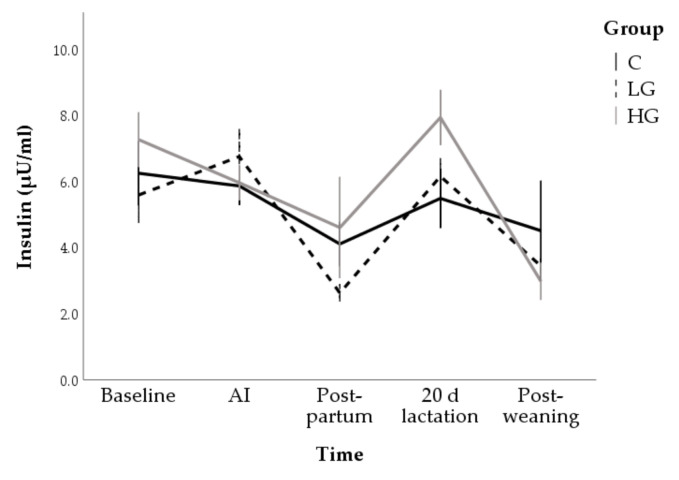
Changes in insulin concentrations during nutrition adaptation and productive cycle of rabbit does. C = control diet; LG = diet supplemented with 1% of goji berries; HG = diet supplemented with 3% of goji berries. AI = artificial insemination.

Overall, insulin concentrations reduced from baseline (6.4 ± 0.4 µU/mL) to post-partum (3.7 ± 0.6 µU/mL; F = 6.71; *p* < 0.001). They increased during lactation (6.5 ± 0.6) but, at weaning, lower concentrations than baseline were found (3.7 ± 0.8 µU/mL; *p* = 0.003). With regards the group effect, only a trend was found at post-partum, when values of LG tended to be lower than HG (*p* = 0.081; Figure 3).

**Figure 4:**
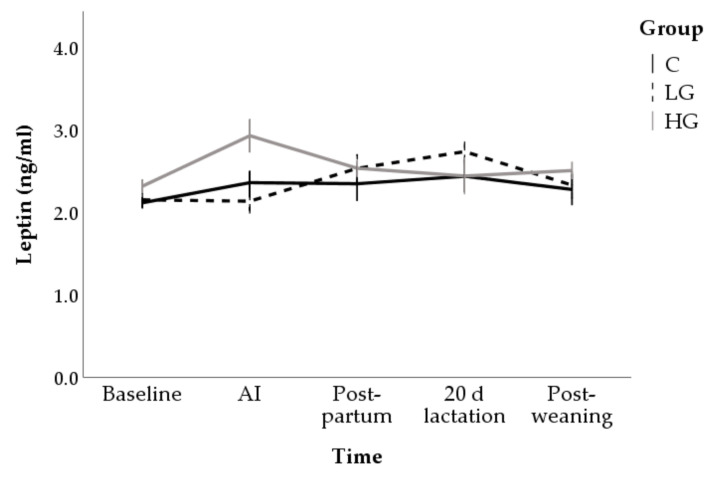
Changes in leptin concentrations during nutrition adaptation and productive cycle of rabbit does. C = control diet; LG = diet supplemented with 1% of goji berries; HG = diet supplemented with 3% of goji berries. AI = artificial insemination.

The HG group showed the highest leptin concentrations at AI (2.9 ± 0.1 ng/mL; *p* < 0.01). Subsequently the concentrations decreased (*p* < 0.05) and returned to baseline values after 20 d of lactation (2.5 ± 0.3 ng/mL). Constant values over time were found in C group (marginal mean: 2.3 ± 0.1 ng/mL) while LG showed greater leptin concentrations during lactation (2.7 ± 0.2 ng/mL) compared to baseline (2.2 ± 0.1 ng/mL; *p* = 0.013; Figure 4).

**Figure 5:**
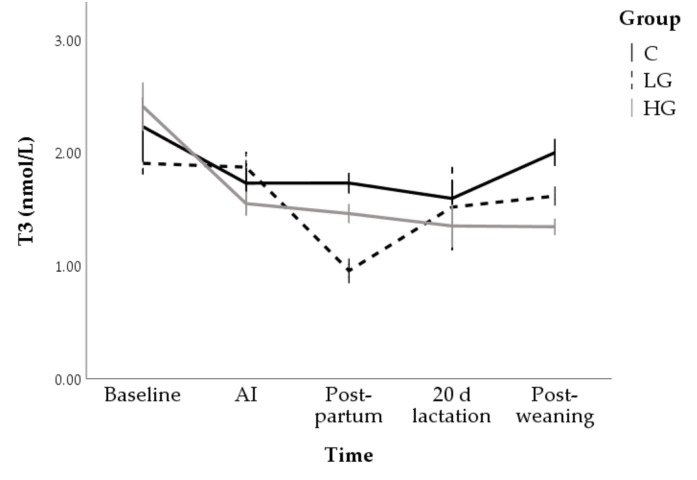
Changes in T3 concentrations during nutrition adaptation and productive cycle of rabbit does. C = control diet; LG = diet supplemented with 1% of goji berries; HG = diet supplemented with 3% of goji berries. AI = artificial insemination.

A progressive reduction until postpartum was observed in T3 concentrations (F = 9.17; *p* < 0.001). This reduction was more marked in the LG group which showed lower marginal means (1.9 ± 0.1 nmol/L and 1.6 ± 0.1 nmol/L in C and LG groups; respectively; *p* < 0.05) and lower postpartum values (1.7 ± 0.2 nmol/L and 1.0 ± 0.2 in C and LG groups, respectively; *p* < 0.05) than the control. After weaning, baseline values were found for both C and LG while HG showed lower T3 concentration (2.4 ± 0.2 nmol/L and 1.3 ± 0.3 nmol/L at time 0 and post weaning, respectively; *p* < 0.01; Figure 5).

**Figure 6:**
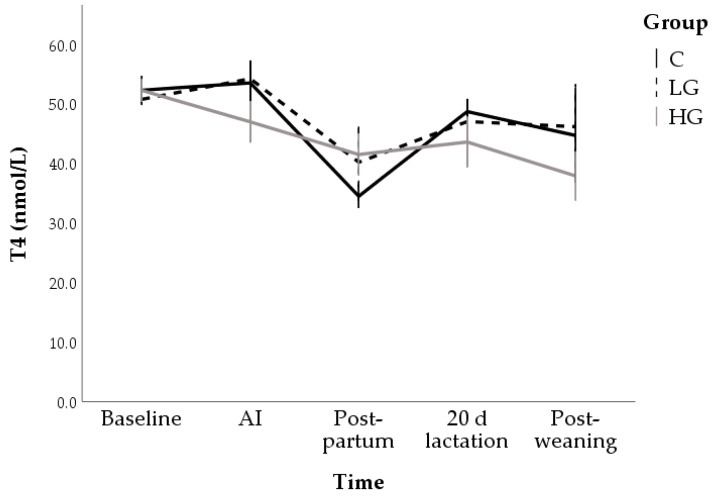
Changes in T4 concentrations during nutrition adaptation and productive cycle of rabbit does. C = control diet; LG = diet supplemented with 1% of goji berries; HG = diet supplemented with 3% of goji berries. AI = artificial insemination.

Only changes over time (F = 5.97; *p* < 0.001) were found for T4 which decreased until post-partum (from 51.8 ± 1.9 nmol/L at T0 to 38.8 ± 2.6 nmol/L at post-partum; *p* < 0.001); subsequently, it increased (46.5 ± 0.2 nmol/L at day 20 of lactation) but its values on the last time, (42.9 ± 3.8 nmol/L) were lower than in the first sampling time (*p* < 0.05). No group-related differences were significant (F = 0.75; *p* = 0.475; Figure 6).

**Figure 7:**
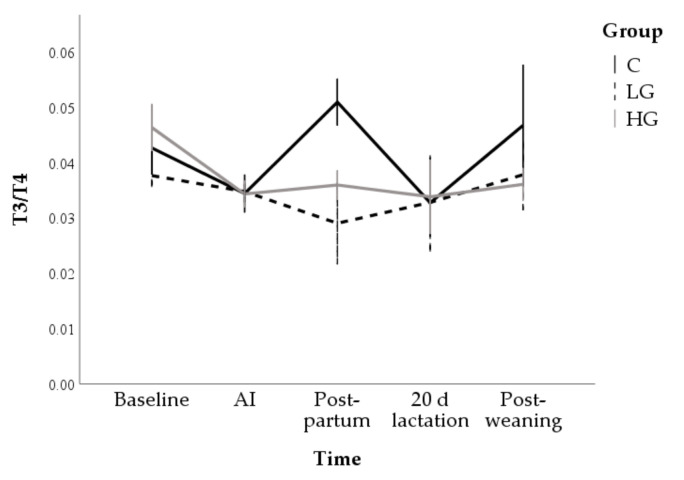
Changes in T3/T4 ratio during nutrition adaptation and productive cycle of rabbit does. C = control diet; LG = diet supplemented with 1% of goji berries; HG = diet supplemented with 3% of goji berries. AI = artificial insemination.

Changes over time in T3/T4 ratio were only found for the Control group (*p* = 0.043) which showed significantly higher values than the LG group at the postpartum (0.034 ± 0.003 and 0.029 ± 0.005 for C and LG, respectively; *p* = 0.010; Figure 7).

**Figure 8:**
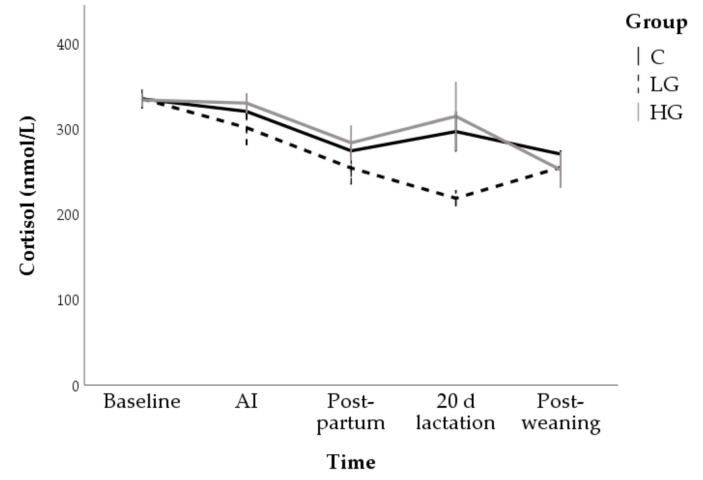
Changes in cortisol concentrations during nutrition adaptation and productive cycle of rabbit does. C = control diet; LG = diet supplemented with 1% of goji berries; HG = diet supplemented with 3% of goji berries. AI = artificial insemination.

Cortisol concentrations progressively decreased in all groups, from 334.3 ± 7.4 nmol/L at baseline to 258 ± 14.4 nmol/L at post-weaning (F = 12.63; *p* < 0.001). The LG group (272.5 ± 7.0 nmol/L; *p* < 0.01) had, on average, the lowest concentrations of cortisol but no multiple comparisons reached statistical significance (Figure 8).

**Figure 9:**
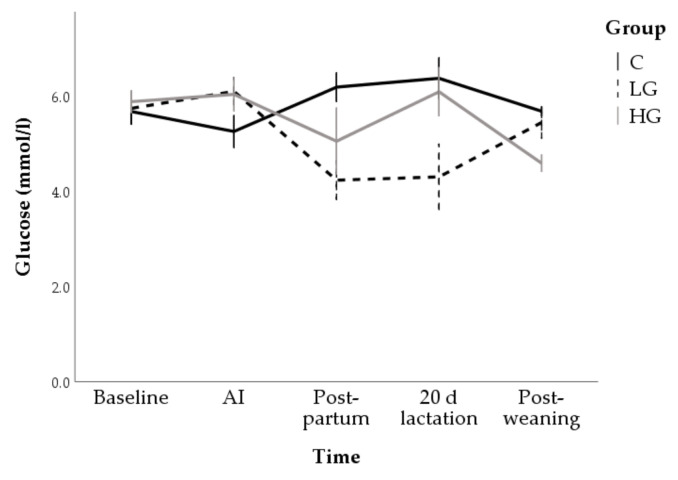
Changes in glucose concentrations during nutrition adaptation and productive cycle of rabbit does. C = control diet; LG = diet supplemented with 1% of goji berries; HG = diet supplemented with 3% of goji berries. AI = artificial insemination.

No change over time was observed in the control group while a reduction in glucose concentrations compared to baseline (5.7 ± 0.4 mmol/L) was observed post-partum (4.2 ± 0.4 mmol/L; *p* < 0.001) and during lactation (4.3 ± 0.4 mmol/L; *p* < 0.001) in the LG group. Moreover, marginal means of LG group (5.5 ± 0.2 mmol/L) was lower than C (5.8 ± 0.2 mmol/L; *p* < 0.05; Figure 9).

**Figure 10:**
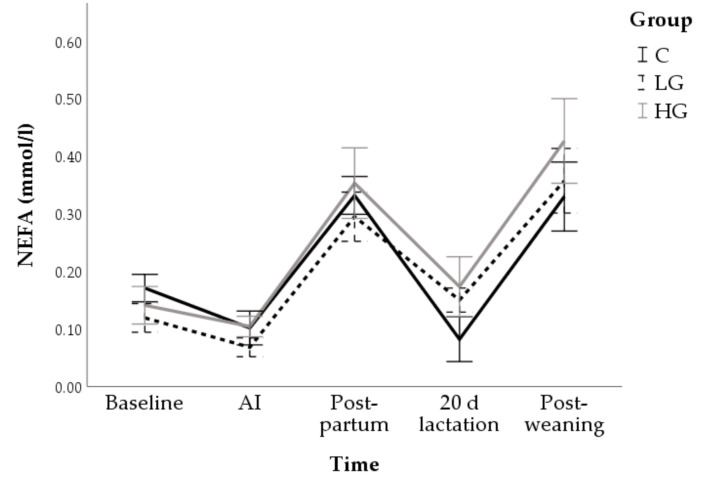
Changes in non-esterified fatty acids (NEFA) concentrations during nutrition adaptation and productive cycle of rabbit does. C = control diet; LG = diet supplemented with 1% of goji berries; HG = diet supplemented with 3% of goji berries. AI = artificial insemination.

Conversely, NEFA showed significant changes over time (F = 30.67; *p* < 0.001) with no difference between groups (F = 1.81; *p* = 0.167). In particular, greater values on their concentration were found at post-partum (0.33 ± 0.02 mmol/L; *p* < 0.001) and post-weaning (0.37 ± 0.03; *p* < 0.001) compared to baseline (0.14 ± 0.02 mmol/L; Figure 10).

**Figure 11:**
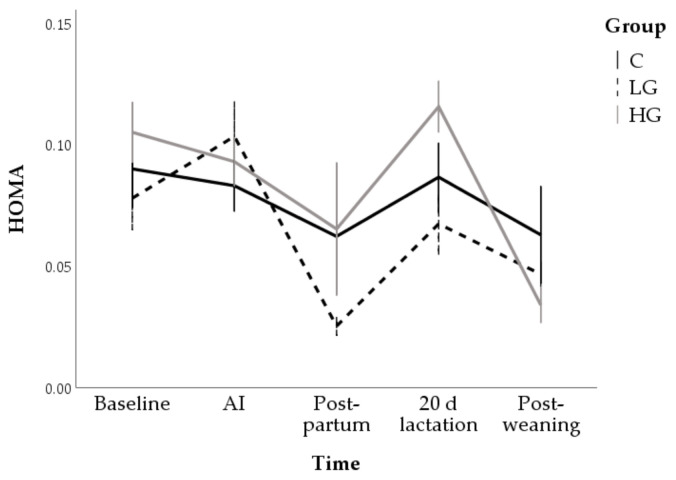
Changes in homeostasis model assessment for insulin resistance index (HOMA) during nutrition adaptation and productive cycle of rabbit does. C = control diet; LG = diet supplemented with 1% of goji berries; HG = diet supplemented with 3% of goji berries. AI = artificial insemination.

Overall, a reduction in HOMA was found at post-partum (0.05 ± 0.01; *p* < 0.01) and post-weaning (0.05 ± 0.01; *p* < 0.01) compared to baseline values (0.09 ± 0.01). This index tended to be lower in LG (0.06 ± 0.01) than HG (0.08 ± 0.01; *p* = 0.063) and, in particular, lower values were found in the post-partum (*p* = 0.072) and at day 20 of lactation (*p* = 0.030; Figure 11).

### 3.2. Multivariable Approach

**Figure 12:**
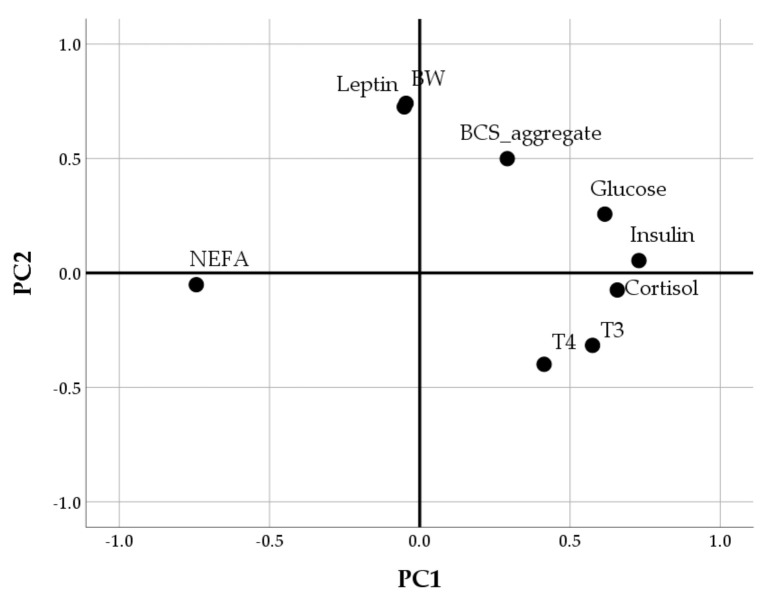
Component plot in rotate space (Varimax rotation). The position of the variables in the Cartesian graph indicates that PC1 (x-axis) was positively associated with insulin, cortisol, glucose, and thyroid hormones while negatively with NEFA levels. The PC2 (y-axis) was positively associated with body weight (BW), leptin, and BCS.

According to the correlation matrix, all the variables indicating body condition and hormonal and metabolic profiles were included in the PCA except for HOMA (Table 2). The Bartlett’s Test of Sphericity (*p* < 0.001) indicated that this dataset was suitable for a data reduction technique and the KMO (=0.70) confirmed the sampling adequacy of the PCA. The PCA extracted two PCs, which together account for 46.1% of the variance (Table 2). The PC1 was a bipolar component including NEFA with negative loading and insulin, cortisol, glucose and thyroid hormones with positive loadings. In the PC2, the highest loadings were found for leptin and body condition indices, BW and BCS (Table 2 and Figure 12).

**Figure 13:**
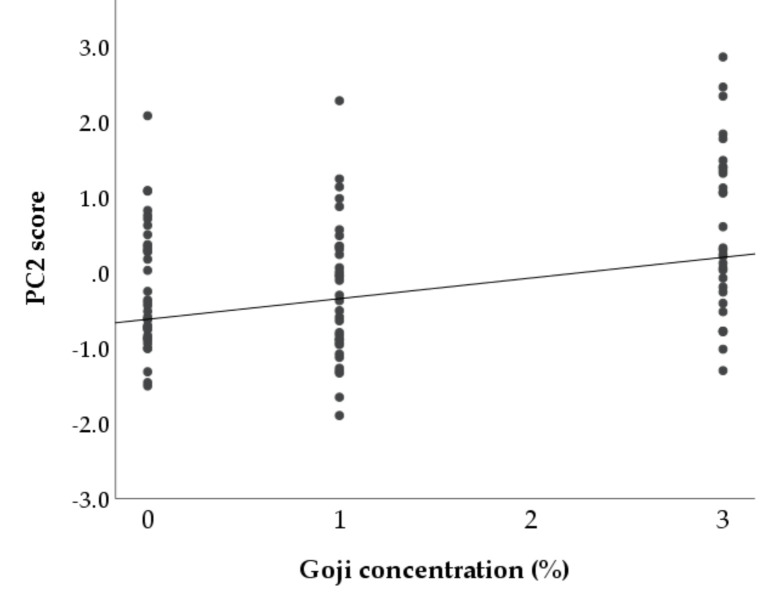
Scatter plot showing the relationship between the percentage of goji berries inclusion in feed (independent variable, x-axis) and PC2 (dependent variable, y-axis). The PC2 was positively associated with body weight, leptin, and BCS. The parameters of regression line were estimated by a generalized linear model including the days of supplementation as covariate.

The two PCs were then analysed by regression techniques to evaluate how the two hormonal-metabolic profiles change as the concentration of supplementation increases (Table 3). Independently from the duration of supplementation, for every 1% increase in the goji concentration, the PC2 score increased by 0.275 (*p* < 0.001). This result indicated that the effects of goji on body conditions and leptin levels are dose-dependent: as the percentage of goji supplementation increases, the BW, the leptin, and the BCS of does tend to increase (Figure 13). In contrast, there was no linear relationship between goji berries inclusion in the diet and PC1 (b = 0.019, *p* > 0.1), which included NEFA, insulin, cortisol, glucose, and thyroid hormones.

Both components changed linearly over time (*p* < 0.01). The signs of b indicated a progressive reduction of PC1 (b = −0.017, *p* < 0.001) while, with lower slope and significance, an increase over time for PC2 (b = 0.007, *p* < 0.01).

## 4. Discussion

Energy homeostasis is finely regulated by many factors which, in a synergistic way, have the task of maintaining a constant body weight. Maintaining this balance is never easy and, in fact, there are frequent alterations that lead to pathological conditions such as obesity and diabetes. During pregnancy, energy homeostasis is even more complicated because the body must also ensure the growth of the foetus and prepare for the next lactation [27,31,32]. The energy balance during a reproduction cycle is one of the most problematic issues for rabbit breeding because it has consequences not only on the animal welfare but also on the profitability of the farm [28,29]. The adoption of new nutritional strategies for the rabbit doe could, therefore, improve both of these aspects. In the present study, a product which is receiving growing interest as a nutraceutical [1,2] and that has shown promising results for the rabbit’s growth performance [15,25] was proposed: the goji berry. Two percentages of supplementation were evaluated in order to find the optimal concentration for goji inclusion in the food of rabbit doe.

The effects of goji supplementation were evaluated on different hormones and metabolites responsible for the maintenance of the energy homeostasis: leptin, insulin, cortisol, thyroid hormones, NEFA, and glucose. All of them are involved in the changes of energy intake, efficiency, and expenditure but the success is only assured if their actions are integrated and strictly coordinated. For this reason, in addition to the description of the individual patterns of these hormones and metabolites, an assessment of the overall picture during the doe’s reproduction cycle was proposed by using multivariate analysis techniques.

In the group fed with standard diet, lower insulin, T3, and cortisol values were found at the end of the production cycle compared to the pre-pregnancy. The T4 remarkably decreased at the post-partum meanwhile the T3/T4 ratio increased. Later, an increase in T4 concentrations was observed but, after weaning, its values were lower than in the first sampling time. Conversely, significant increases were observed for NEFA concentrations with peaks at the post-partum and post-weaning. The concentrations of leptin and glucose did not undergo great changes in the timing of collection. Finally, HOMA insulin sensitivity index showed a fluctuating trend but, at the end of the observation period, its values had decreased.

These hormones and metabolites have recently been evaluated by Menchetti et al. [31] in pregnant and pseudopregnant rabbits. However, this previous study focused on changes during the 32 days post insemination, the sampling was weekly, and no observations were done after the birth. By using weekly sampling, these authors highlighted short-term changes during pregnancy, especially in insulin and leptin. These changes suggested a condition of insulin and leptin resistance that characterizes the pregnancy of many mammals including women [30,32,42,43,44,45]. In the present study, the changes were assessed at longer intervals but the concentrations at early and late pregnancy were comparable to those of Menchetti et al. [31]. Moreover, the longer-term evaluations of the present study allowed to highlight the physiological conditions of the lactating rabbits. In particular, the post-partum increases in NEFA and insulin resistance confirmed the state of negative energy balance during early lactation. In this phase, in fact, there is the big gap between the energy the energy required for maintenance and milk production and that taken with the diet. Several factors can contribute to this severe energy deficit of the does, such as its prolificacy, the quantity and characteristics of the milk, and the intense reproductive rhythm [29,46].

This state is characterized by an intense mobilization of the body reserves which increase the NEFA concentrations. At the same time, the increase in insulin was associated with moderate concentrations of glucose triggering a peripheral insulin resistance status [47] which could contribute to raise NEFA levels. Insulin is, indeed, an anti-lipolytic hormone and the insulin resistance can favor lipolysis and fatty acid availability.

The condition of insulin resistance was exacerbated when the highest goji supplementation was included in the feed (3%, HG group). A similar condition has been found in lactating dairy cows consuming excessive concentrate [47,48]. The insulin resistance develops as a homeorhetic adaptation to ensure a sufficient glucose supply for the lactating mammary gland [49] but it has been negatively associated with milk production, reproduction performance, and animal health [50,51]. The insulin resistance observed in the present study could therefore explain the low milk yields reported by Menchetti et al. [15] in rabbits receiving 3% of goji supplementation. Goji berries contain high amounts of polysaccharides and polyphenols [1]. Although most of the studies reported that these compounds exert beneficial effects on metabolic diseases [4,8,52], their excessive consumption during pregnancy and lactation is debated. For example, Caimari et al. [53] demonstrated that supplementations with a grape seed extract (rich in polyphenols) in pregnant and lactating rats induced insulin and adiponectin resistance. On the other hand, other authors report several positive effects of the administration of polyphenols in lactation: they seem to increase the immunoglobulins content in colostrum of sows and improve the preweaning survivability [54], to protect bovine mammary epithelial cells from oxidative stress [55] and also to exert positive effects on the adult offspring’s metabolism [56,57].

Thus, our findings suggest that the quantity and source of energy during lactation affect milk yield in rabbit doe by modulating body mobilization and insulin sensitivity. Moreover, they raise concerns about the excessive consumption of polyphenol-enriched foods during lactation although dose, source, species, and duration of polyphenols administration should be evaluated from time to time.

Menchetti et al. [15] also reported greater BWs in rabbits fed with the 3% of goji. In the present study, a greater BCS was found in the same group of does. This could suggest that high-doses of Goji supplementation also leads to excessive fattening which, in rabbit does, is negatively associated with reproductive performance [58].

The effects of a lower supplementation diverged: the does supplemented with 1% goji showed lower T3 at post-partum as well as lower cortisol levels during lactation; their leptin concentrations were lower at AI and higher during lactation while insulin, glucose and HOMA values suggested a higher insulin sensitivity during lactation. This hormonal framework would seem to favour the milk yield as high productions were recorded by Menchetti et al. [15] with the low-dose goji supplementation. For example, the low levels of T3 at the beginning of lactation could decrease thermogenesis diverting nutrients to the mammary glands. As mentioned above, the positive association between insulin sensitivity and lactation has been already showed in ruminants and humans [59]. These findings suggest that the effects of goji berries on insulin resistance are dose-dependent and a moderate consumption could improve insulin sensitivity during the reproductive cycle.

The increase in leptin concentrations during lactation could explain the differences in food intake between groups as this is the main function of leptin, as well as the most studied [60]. Some authors reported hypoleptinemia during lactation and have hypothesized that leptin resistance may also continue after giving birth [61,62]. However, our results do not seem to support these theses. On the other hand, maternal leptin circulating during lactation may be important for the neurological development of the new-borns because it is secreted into milk and it is taken up during suckling [63]. As observed in several species including humans, the leptin ingested trough suckling could have a role not only in the changes during the perinatal period but also in the metabolic programming of adulthood phenotype [64,65]. Finally, Koch et al. [66] demonstrated that rabbit mammary tissue expresses leptin mRNA and protein during lactation suggesting that it could be also involved in the mammary gland development.

In the present study, all the variables described so far were then included in the multivariate analysis to identify the hormonal and metabolic profiles characterizing rabbit pregnancy and lactation. Two dimensions were extracted.

The conditions of insulin resistance and energy deficit were well described by the first component extracted by the principal component analysis. In this component, insulin and glucose had the same sign (both positive loadings) and this could be explained by the reduction in insulin sensitivity: insulin rises as the glucose increases but, under conditions of insulin resistance, cells fail to respond normally and high blood glucose levels are still maintained. In the first dimension, instead, the insulin was opposed to NEFA levels. The anti-lipolytic effect of insulin could explain the opposite signs of these items. Indeed, the known effect of systemic insulinization is a decline in circulating NEFA concentrations [67]. However, rabbits’ pregnancy and lactation are characterized by elevated NEFA levels. To explain the negative association between NEFA and insulin levels in this context, it can also be hypothesized that chronically elevated NEFA levels reduce the insulin-secretory capacity in rabbit does, as already proposed for high-yielding dairy cows [51].

Overall, the first dimension progressively reduced, suggesting that insulin, cortisol, glucose, and thyroid hormones decreased during the productive cycle of the doe while, in the meantime, the NEFAs increased. Similar profiles are described in lactating cows [47].

The second dimension extracted by the principal component analysis included leptin, a hormone secreted mainly by adipose tissue [60]. Therefore, its positive associations with body weight and BCS found in this component are not surprising. The positive loadings of glucose and insulin in this dimension also confirm that they could be factors implicated in leptin regulation [60]. Of major interest is the positive relationship between this second component and goji supplementation indicating that leptin, BW and BCS enhance as the percentage of goji berries inclusion in the diet increases. In agreement with the univariate results, this finding suggests that there is a risk of excessive fattening in rabbit does supplemented with goji and that, probably, a high percentage of inclusion ultimately determines detrimental reproductive effects.

It is worthwhile noting that 3% of goji supplementation had a different impact in growing rabbits: it guaranteed good growth performance and an improvement in the meat quality [15,25]. The use of goji in rabbit diet was also positively perceived by consumers who probably appreciate the use of nutraceutical products also in animal nutrition [25]. Overall, the goji inclusion in the feed could be a promising nutritional strategy from both a marketing and animal welfare point of view. However, the dosage of goji berries should be differentiated according to the category of animals and physiological status.

## 5. Conclusions

The rabbit doe is subjected to a severe energy deficit during reproductive cycle so that several physiological phenomena, such as the increase in NEFA and insulin resistance, are comparable to those of lactating cows. The quantity and source of energy during pregnancy and lactation modulate body mobilization, insulin sensitivity and leptin levels that, in turn, could affect milk yield. This balance is so delicate that the effects of goji supplementation diverged according to the dose: a moderate consumption seems improve insulin resistance while a high-dose could make animals excessively fat and reduce insulin sensitivity. This work has proved that goji berry could play a role in rabbit nutrition, but the formulation should be carefully modulated according to the physiological state.

## Figures and Tables

**Table 1:** Formulation and chemical composition (as fed) of control (C) and experimental diets supplemented with goji berries. LG = diet supplemented with 1% of goji berries; HG = diet supplemented with 3% of goji berries.

Parameter	Unit	Diet		
		C	LG	HG
**Ingredients**				
Wheat bran	%	30.0	29.5	29.0
Dehydrated alfalfa meal	%	42.0	41.5	41.0
Barley	%	9.5	9.5	9.0
Sunflower meal	%	4.5	4.5	4.2
Rice bran	%	4.0	4.0	3.9
Soybean meal	%	4.0	4.0	3.9
Calcium carbonate	%	2.2	2.2	2.2
Cane molasses	%	2.0	2.0	2.0
Vitamin-mineral premix *	%	0.4	0.4	0.4
Soybean oil	%	0.4	0.4	0.4
Salt	%	0.3	0.3	0.3
Goji berries	%	-	1.0	3.0
**Analytical data**				
Crude Protein	%	15.74	15.64	15.66
Ether extract	%	2.25	2.23	2.47
Ash	%	9.28	9.36	9.25
Starch	%	16.86	17.07	16.99
NDF	%	38.05	38.55	37.49
ADF	%	19.54	19.60	19.01
ADL	%	4.01	4.31	3.98
**Digestible Energy** **	Kcal/kg	2464	2463	2459

* Per kg diet: vitamin A 11,000 IU; vitamin D3 2000 IU; vitamin B1 2.5 mg; vitamin B2 4 mg; vitamin B6 1.25 mg; vitamin B12 0.01 mg; alpha-tocopherol acetate 50 mg; biotine 0.06 mg; vitamin K 2.5 mg; niacin 15 mg; folic acid 0.30 mg; D-pantothenic acid 10 mg; choline 600 mg; Mn 60 mg; Fe 50 mg; Zn 15 mg; I 0.5 mg; Co 0.5 mg. ** Estimated by Maertens et al. [34].

**Table 2:** Loadings of factors extracted with the principal component analysis.

Item	Component	
	PC1	PC2
**NEFA**	**−0.744**	−0.051
**Insulin**	**0.729**	0.054
**Cortisol**	**0.658**	−0.075
**Glucose**	**0.616**	0.257
**T3**	**0.575**	−0.316
**T4**	**0.414**	−0.399
**BW**	−0.046	**0.741**
**Leptin**	−0.052	**0.725**
**BCS**	0.291	**0.499**
**% Variance explained**	27.7%	18.4%
**Cumulative % variance explained**	46.1%	

Factor loadings with an absolute value greater than 0.4 are in bold. NEFA = non-esterified fatty acids; T3 = triiodothyronine; T4 = thyroxine; BW = body weight; BCS = Body Condition Score.

**Table 3:** Results of regression analyses including percentage of goji berries inclusion in feed as independent variables and principal component as outcome. Days of supplementation were included as covariate.

Dependent Variable/Outcome	Independent Variable/Predictor	B Coefficient	Standard Error	*p* Value
**PC1**	**Constant**	0.611	0.1511	<0.001
	**Goji berries concentration in feed**	0.019	0.0663	0.776
	**Days of supplementation**	−0.017	0.0024	<0.001
**PC2**	**Constant**	−0.619	0.1373	<0.001
	**Goji berries concentration in feed**	0.275	0.0765	<0.001
	**Days of supplementation**	0.007	0.0027	0.006
